# Dynamics of a stochastic COVID-19 epidemic model with jump-diffusion

**DOI:** 10.1186/s13662-021-03396-8

**Published:** 2021-05-01

**Authors:** Almaz Tesfay, Tareq Saeed, Anwar Zeb, Daniel Tesfay, Anas Khalaf, James Brannan

**Affiliations:** 1 School of Mathematics and Statistics & Center for Mathematical Sciences, Huazhong University of Science and Technology, Wuhan, 430074 China; 2 Department of Mathematics, Mekelle University, P.O. Box 231, Mekelle, Ethiopia; 3 Department of Mathematics, King Abdulaziz University, Jeddah, 41206 Kingdom of Saudi Arabia; 4 Department of Mathematics, COMSATS University Islamabad, Abbottabad Campus, Abbottabad, 22060 Khyber Pakhtunkhwa Pakistan; 5 Department of Mathematical Sciences, Clemson University, Clemson, South Carolina 29634 USA

**Keywords:** 39A50, 45K05, 65N22, Brownian motion, Lévy noise, Stochastic COVID-19 model, Extinction, Persistence

## Abstract

For a stochastic COVID-19 model with jump-diffusion, we prove the existence and uniqueness of the global positive solution. We also investigate some conditions for the extinction and persistence of the disease. We calculate the threshold of the stochastic epidemic system which determines the extinction or permanence of the disease at different intensities of the stochastic noises. This threshold is denoted by *ξ* which depends on white and jump noises. The effects of these noises on the dynamics of the model are studied. The numerical experiments show that the random perturbation introduced in the stochastic model suppresses disease outbreak as compared to its deterministic counterpart. In other words, the impact of the noises on the extinction and persistence is high. When the noise is large or small, our numerical findings show that COVID-19 vanishes from the population if $\xi <1$; whereas the epidemic cannot go out of control if $\xi >1$. From this, we observe that white noise and jump noise have a significant effect on the spread of COVID-19 infection, i.e., we can conclude that the stochastic model is more realistic than the deterministic one. Finally, to illustrate this phenomenon, we put some numerical simulations.

## Introduction

Infectious diseases are the public enemy of the human population and have brought a great impact on the mankind. In the present time, the novel coronavirus is the major disease in the world. This new strain of coronavirus is called COVID-19 or SARS-Cov2. COVID-19 has been declared by the World Health Organization as a global emergency in January 2020 and a pandemic in March 2020 [1]. Since the first breakout of the pandemic, according to the data released by World barometer [2], there have been more than 52 million confirmed (from which 17 million are active) cases, 1.29 million deaths, and 33.5 million recoveries from the disease.

Researchers are working around the clock to gain in-depth understanding of the nature of the disease. Scientists are also battling to produce a vaccine for this new virus.

Numerous scholars have conducted investigation to predict the spread of COVID-19 in order to seek the best prevention measures. For example, [3–8] studied mathematical models of COVID-19 to describe the spread of the coronavirus. Stochastic transition models were established in [9–11] to evaluate the spread of COVID-19. The importance of isolation and quarantine was also emphasized in those articles. Dalal et al. [12] studied the impact of the environment on the AIDS model using the method of parameter perturbation. In papers [13–20] fractal-fractional differentiation and integration was discussed. This approach is very important in investigating the stochastic COVID-19 model.

Stochastic dynamical systems are widely used to describe different complex phenomena. The random fluctuations in complex phenomena usually portray intermittent jumps, i.e., the noises are non-Gaussian. In other words, epidemic models are inevitably subject to environmental noise, and it is necessary to reveal how the environmental noise influences the epidemic model. In the natural world, there are different types of random noises, such as the well-known white noise, the Lévy jump noise which considers the motivation that the continuity of solutions may be inevitably under severe environmental perturbations, such as earthquakes, floods, volcanic eruptions, SARS, influenza [21–23], and a jump process should be introduced to prevent and control diseases, and so on. Mathematically, several authors [24–27] used the Lévy process to describe the phenomena that cause a big jump to occur occasionally.

Recently, Zhang et al. [28] investigated the stochastic COVID-19 mathematical model driven by Gaussian noise. The authors assumed environmental fluctuations in the constant *β*, so that $\beta \longrightarrow \beta +\lambda \dot{B}_{t}$, where $B_{t}$ is a one-dimensional Brownian motion [29]. The stochastic COVID-19 model which they considered is

1 $$\begin{aligned} &dS_{t}= (\Lambda -\beta S_{t} I_{t}-\nu S_{t}+\sigma R_{t})\,dt- \lambda S_{t} I_{t} \,dB_{t}, \\ &dI_{t}=\bigl( \beta S_{t} I_{t}-(\nu +\gamma ) I_{t}\bigr)\,dt+\lambda S_{t} I_{t} \,dB_{t}, \\ &dR_{t}=\bigl(\gamma I_{t}-(\nu +\sigma ) R_{t} \bigr)\,dt, \end{aligned}$$

where the variables $S_{t}, I_{t}$, and $R_{t}$ represent the susceptible population, infectious population, and recovered (removed) population, respectively. The parameters $\Lambda, \beta, \nu, \gamma $, and *σ* are all positive constant numbers, and they represent the joining rate of the population to susceptible class through birth or migration, the rate at which the susceptible tend to infected class (like social distancing $\beta \in (0,1)$), due to a natural cause and from COVID-19, the recovery rate, and the rate of health deterioration, respectively. $B_{t}$ is the standard Brownian motion defined on the complete probability space $(\Omega, \mathcal{F},\{\mathcal{F}_{t}\}_{t\geq 0}, \mathbb{P})$ and *λ* is the intensity of the Gaussian noise. The researchers proved the existence and uniqueness of the nonnegative solution of system (1), and they also showed the extinction and persistence of the disease. But they did not consider the jump noise.

Since the stochastic model (1) that does not take randomness cannot efficiently model these phenomena, the Lévy noise, which is more comprehensive, is a better candidate [30].

Here, we consider that the environmental Gaussian and non-Gaussian noises are directly proportional to the state variables $S_{t}, I_{t}$, and $R_{t}$. Several scholars used this approach, for instance, we refer to [31–33] and the references therein. The system which we consider has the following form:

2 $$\begin{aligned} &dS_{t}= (\Lambda -\beta S_{t-} I_{t-}-\nu S_{t-}+\sigma R_{t-}) \,dt+\lambda _{1} S_{t-} \,dB^{1}_{t} + \int _{\mathbb{Y}} \epsilon _{1}(y)S_{t-} \bar{N}(dt,dy), \\ &dI_{t}=\bigl( \beta S_{t-} I_{t-}-(\nu +\gamma ) I_{t-}\bigr) \,dt+\lambda _{2} I_{t-} \,dB^{2}_{t}+ \int _{\mathbb{Y}} \epsilon _{2}(y)I_{t-} \bar{N}(dt,dy), \\ &dR_{t}=\bigl(\gamma I_{t-}-(\nu +\sigma ) R_{t-} \bigr) \,dt+\lambda _{3} R_{t-} \,dB^{3}_{t}+ \int _{\mathbb{Y}} \epsilon _{3}(y)R_{t-} \bar{N}(dt,dy), \end{aligned}$$

where $S_{t-}$ is the left limit of $S_{t}$. The description of the parameters $\Lambda, \beta, \nu, \gamma $, and *σ* are the same as in model (1). For $j=1,2,3$, $\epsilon _{j}(y)$ is a bounded function satisfying $\epsilon _{j}(y)+1>0$ on the intervals $|y|\geq 1$ or $|y|<1$. $N(t,dy)$ is the independent Poisson random measure on $\mathbb{R}^{+}\times {{\mathbb{R}}\setminus {\{0\}}}$, $\bar{N}(t,dy)$ is the compensated Poisson random measure satisfying $\bar{N}(t,dy)=N(t,dy)-\pi (dy)\,dt$, where $\pi (.)$ is a *δ*-finite measure on a measurable subset $\mathbb{Y}$ of $(0,\infty )$ and $\pi (\mathbb{Y})<\infty $ [30, 34]. $B^{j}_{t}$ is mutually independent standard Brownian motion and $\lambda _{j}$ stands for the intensities of the Gaussian noise [35]. To the best of our knowledge, this model is not studied before.

In this study, we are going to investigate the stochastic COVID-19 model with jump-diffusion (2). The existence of the solution of the stochastic model (2) is analyzed. We use the Euler–Maruyama (EM) method, which was proposed in [36, 37], after revising and changing it a bit to fit our model. The consistency, convergence, and stability of this numerical method is also proved in the afore-mentioned papers. This method helps to evaluate explanations based on the notion of adversarial robustness. Using numerical simulations, we study the impact of the deterministic parameters and noise intensities on the proposed system. We think this is a better tool to demonstrate the interactions between the epidemic system and its complex surrounding. Especially, we focus on the extinction and persistence of the SARS-Cov2 and present the biological interpretations. The evaluation criteria further allow us to derive new explanations which capture pertinent features qualitatively and quantitatively. From the plotted figures, we can observe that the noise intensities have a great impact on systems (4) and (2). More details are given in Sects. 4 and 5.

The goal of the present work is to make contributions to understanding the dynamics of the novel disease (COVID-19) epidemic models with both Gaussian and non-Gaussian noises, i.e., we aspire to study the effect of Gaussian noise and jumps intensities on COVID-19 epidemic.

The rest of the paper is constituted as follows. In Sect. 2, we recall some important notations and lemmas. In Sect. 3, we discuss the dynamical behavior of the deterministic COVID-19 model. Section 4 has two subsections. The existence and uniqueness of the solution of the stochastic COVID-19 model (2) is given in Sect. 4.1; while in Sect. 4.2, by finding the value of the threshold, we show the conditions for the extinction and persistence to COVID-19. The discussion and numerical experiments of our work are given in Sect. 5. Finally, we present conclusion of our study in Sect. 6.

## Preliminaries

In this section, we introduce some basic notations and lemmas. Throughout this paper, we have

- $(\Omega, \mathcal{F},\{\mathcal{F}_{t}\}_{t\geq 0}, \mathbb{P})$ denotes a complete filtered probability space;
- $\mathbb{R}^{3}_{+}:=\{x=(x_{1},x_{2},x_{3})\in \mathbb{R}^{3}: x_{j} \geq 0, j=1,2,3\}$, $\mathbb{R}_{+}=(0,\infty )$;
- For the jump-diffusion, let $n\geq 0$, there is a positive constant $L_{n}$ such that
  (i.) $\int _{\mathbb{Y}} |H_{j}(x,y)-H_{j}(\bar{x},y)|^{2} \pi (dy) \leq L_{n} |x-\bar{x}|^{2}$, where $H_{j}(x,y)=\epsilon _{j}(y) X_{t}, j=1,2,3$. For more details, we refer to [38, p. 78], [39];
  (ii.) $1+\epsilon _{j}(y)\geq 0, y\in \mathbb{Y}, j=1,2,3$, there exists $C > 0$ such that $\int _{\mathbb{Y}} (\ln (1+\epsilon _{j}(y)))^{2} \pi (dy) < C$;
- $\langle M \rangle_{t}=\frac{1}{t}\int _{0}^{t}M_{r}\,dr$, $\langle M \rangle^{*}_{t}=\lim_{t\rightarrow \infty }\inf \frac{1}{t}\int _{0}^{t}M_{r}\,dr$, $\langle M \rangle_{t}^{**}=\lim_{t\rightarrow \infty }\sup \frac{1}{t}\int _{0}^{t}M_{r}\,dr$;
- For $j=1,2,3$, $\varphi _{j}=\frac{\lambda ^{2}_{j}}{2}+\int _{\mathbb{Y}} ( \epsilon _{j}(y)-\ln (1+\epsilon _{j}(y))) \pi (dy), j=1,2,3$;
- $\psi _{j}=\int _{\mathbb{Y}} (\ln (1+\epsilon _{j}(y))) \bar{N}(dt,dy)$, $<\psi _{j},\psi _{j}>=t\int _{\mathbb{Y}} (\ln (1+\epsilon _{j}(y))) \pi (dy)<t C$;
- For some positive $m>2$, $M=\nu -\frac{m-1}{2} \bar{\Lambda }^{2}-\frac{1}{m} \bar{\epsilon }$, where $\bar{\Lambda }=\max \{\lambda _{1}^{2}, \lambda _{2}^{2}, \lambda _{3}^{2} \}$, and $\bar{\epsilon }=\int _{\mathbb{Y}}(1+\tilde{\epsilon })^{m}-1-m \hat{\epsilon } \pi (dy)$, where $\tilde{\epsilon }=\max \{\epsilon _{1}(y), \epsilon _{2}(y), \epsilon _{3}(y) \}$, and $\hat{\epsilon }=\min \{\epsilon _{1}(y),\epsilon _{2}(y),\epsilon _{3}(y) \}$;
- $\inf \emptyset =\infty $, where ∅ denotes an empty set.

### Remark 1

For some positive *x*, the following is true: $x-1-\ln x >0$.

### Lemma 1

(The one-dimensional Itô formula)

*Here we will give Itô’s formula for the following* *n*-*dimensional stochastic differential equation* (*SDE*) *with jump noise* [30]:

3 $$\begin{aligned} dY(t)=G\bigl(Y(t)\bigr)\,dt+F\bigl(Y(t)\bigr)\,dB_{t}+ \int _{|y|< 1}H\bigl(Y(t),y\bigr)\bar{N}(dt,dy),\quad t\geq 0, \end{aligned}$$

*where* $G:\mathbb{R}_{+}\times \mathbb{R}^{n}\rightarrow \mathbb{R}^{n}$, $F:\mathbb{R}_{+}\times \mathbb{R}^{n}\rightarrow \mathbb{R}^{n} \times \mathbb{R}^{d}$, $H:\mathbb{R}_{+}\times \mathbb{R}^{n}\times \mathbb{R}^{n} \rightarrow \mathbb{R}^{n}$ *for* $n\geq 2$ *are considered as measurable*.

Assume *Y* to be a solution of SDE (3). Then, for each $W \in C^{2}(\mathbb{R}^{n})$, $t\in [0,\infty )$, with probability one, we have [38]

$$\begin{aligned} &W\bigl(Y(t)\bigr)-W\bigl(Y(0)\bigr) \\ &\quad= \int _{0}^{t} \partial _{j}W \bigl(Y_{c}\bigl(r^{-}\bigr)\bigr)\,dY^{j} + \frac{1}{2} \int _{0}^{t}\partial _{j}\partial _{i}W\bigl(Y_{c}\bigl(r^{-}\bigr)\bigr)\,d \bigl[Y^{j}_{c},Y^{i}_{c}\bigr](r) \\ &\qquad{}+ \int _{0}^{t} \int _{|y|< 1}\bigl[W\bigl(Y\bigl(r^{-}\bigr)+H \bigl(Y(r),y\bigr)\bigr)-W\bigl(Y\bigl(r^{-}\bigr)\bigr)\bigr] \bar{N}(dr,dy) \\ &\qquad{}+ \int _{0}^{t} \int _{|y|< 1}\bigl[W\bigl(Y\bigl(r^{-}\bigr)+H \bigl(Y(r),y\bigr)\bigr)-W\bigl(Y\bigl(r^{-}\bigr)\bigr)\\ &\qquad{}-H^{i} \bigl(Y(r),y\bigr) \partial _{i}W\bigl(Y\bigl(r^{-}\bigr) \bigr)\bigr]\pi (dy)\,dr, \end{aligned}$$

where $Y_{c}$ is the continuous part of *Y* given by $Y^{i}_{c}(t)=\int _{0}^{t}F^{i}_{k}(s)\,dB^{k}(s)+\int ^{t}_{0}G^{i}(s)\,ds$, $1\leq i\leq n, 1\geq k\leq m, t\geq 0$. The proof of this lemma is given in [30, p. 226].

Next, let us denote by $LW:[0,\infty )\times \mathbb{R}^{n}\rightarrow \mathbb{R}$ the linear function associated with SDE (3) which is given by

$$\begin{aligned} (LW) (\eta )={}& G^{i}(\eta ) (\partial _{i}W) \bigl(\eta (0)\bigr)+\frac{1}{2}\bigl[F( \eta ) (F(\eta )^{T} \bigr]^{ik}(\partial _{i}\partial _{k}W) \bigl( \eta (0)\bigr) \\ &{}+ \int _{|y|< 1}\bigl[W\bigl(\eta (0)+H(\eta,y)\bigr)-W\bigl(\eta (0) \bigr)-H^{i}(\eta,y) ( \partial _{i}W) \bigl(\eta (0)\bigr) \bigr]\pi (dy), \end{aligned}$$

where $\eta \in [0,\infty )\times \mathbb{R}^{n} $.

### Lemma 2

*Assume that* $(c)$ *holds*. *The stochastic model* (2) *has a unique nonnegative solution* $(S_{t},I_{t},R_{t})\in \mathbb{R}_{+}^{3}$ *for any given initial value* $(S_{0},I_{0},R_{0})\in \mathbb{R}^{3}_{+}$ *on time* $t\geq 0$ *almost surely* (*a*.*s*.). *Under* $(g)$, *the solution of model* (2) *satisfies the following conditions*:

(i.) $\lim_{t \rightarrow \infty } (\frac{S_{t}+I_{t}+R_{t}}{t} )=0$ *a*.*s*.

*Moreover*, $\lim_{t \rightarrow \infty } (\frac{S_{t}}{t} )=0, \lim_{t \rightarrow \infty } (\frac{I_{t}}{t} )=0, \lim_{t \rightarrow \infty } (\frac{R_{t}}{t} )=0$;

(ii.) $\lim_{t \rightarrow \infty }\frac{S_{t}\,dB^{1}_{t}}{t}=0$, $\lim_{t \rightarrow \infty }\frac{I_{t}\,dB^{2}_{t}}{t}=0$, $\lim_{t \rightarrow \infty }\frac{R_{t}\,dB^{3}_{t}}{t}=0$, $\lim_{t \rightarrow \infty } \frac{\int _{0}^{t} \int _{\mathbb{Y}}S_{r} \epsilon _{1}(y) \bar{N}(dr,dy)}{t}=0$, $\lim_{t \rightarrow \infty } \frac{\int _{0}^{t} \int _{\mathbb{Y}}I_{r} \epsilon _{2}(y) \bar{N}(dr,dy)}{t}=0$, $\lim_{t \rightarrow \infty } \frac{\int _{0}^{t} \int _{\mathbb{Y}}R_{r} \epsilon _{3}(y) \bar{N}(dr,dy)}{t}=0$. *a*.*s*.

### Proof

The proof of this lemma is similar to [33] and hence is omitted. □

## Dynamical analysis of the deterministic COVID-19 model

The deterministic version of systems (1) and (2) is

4 $$\begin{aligned} &\frac{dS_{t}}{dt}= \Lambda -\beta S_{t} I_{t}-\nu S_{t}+\sigma R_{t}, \\ &\frac{dI_{t}}{dt}=\beta S_{t} I_{t}-(\nu +\gamma ) I_{t}, \\ &\frac{dR_{t}}{dt}t=\gamma I_{t}-(\nu +\sigma ) R_{t}, \end{aligned}$$

and

5 $$\begin{aligned} \frac{dX}{dt}=\frac{dS_{t}}{dt}+\frac{dI_{t}}{dt}+ \frac{dR_{t}}{dt}= \Lambda -\nu X, \end{aligned}$$

where $X=S_{t}+I_{t}+R_{t}$. For $\Lambda =\nu X$, equation (5) shows that *X* is the total constant population with the initial value $X_{0}=S_{0}+I_{0}+R_{0}$. This equation has analytical solution

6 $$\begin{aligned} X=\frac{\Lambda }{\nu }+X_{0} e^{-\nu t}. \end{aligned}$$

Since the initial values are nonnegative, we have $S_{t}\geq 0, I_{t}\geq 0, R_{t}\geq 0$, and $\lim_{t\rightarrow \infty }X=\frac{\Lambda }{\nu }$. One can easily conclude that $0 < X \leq \frac{\Lambda }{\nu }$. Therefore, Eq. (6) has a positivity property. Thus the deterministic COVID-19 model (4) is biologically meaningful and bounded in the domain

$$ \mathbb{D}= \biggl\{ (S_{t},I_{t},R_{t})\in \mathbb{R}^{3}_{+}: 0 < X \leq \frac{\Lambda }{\nu } \biggr\} . $$

The equilibrium point of system (4) satisfies the following:

$$\begin{aligned} & \Lambda -\beta S_{t} I_{t}-\nu S_{t}+\sigma R_{t}=0, \\ &\beta S_{t} I_{t}-(\nu +\gamma ) I_{t}=0, \\ &\gamma I_{t}-(\nu +\sigma ) R_{t}=0, \end{aligned}$$

having the equilibria:

$$\begin{aligned} &E^{0}=\bigl(S^{0},I^{0},R^{0} \bigr)= \biggl(\frac{\Lambda }{\nu },0,0 \biggr) , \\ &E^{1}=\bigl(S^{1},I^{1},R^{1}\bigr)= \biggl(\frac{\nu +\gamma }{\beta }, \frac{\beta \Lambda -\nu (\nu +\gamma )}{\nu +\gamma },0 \biggr) , \\ & E^{2}=\bigl(S^{2},I^{2},R^{2}\bigr)= \biggl(\frac{\nu +\gamma }{\beta }, 0, \frac{\nu (\nu +\gamma )-\beta \Lambda }{\beta \sigma } \biggr) , \\ & E^{3}=\bigl(S^{3},I^{3},R^{3}\bigr)= \biggl(\frac{\nu +\gamma }{\beta }, \frac{(\Lambda -\nu S^{3})(\nu +\sigma )}{\beta S^{3}(\nu +\sigma )-\gamma \sigma }, \frac{\gamma (\Lambda -\nu S^{3})}{\beta S^{3}(\nu +\sigma )-\gamma \sigma } \biggr), \end{aligned}$$

where $S^{3}=\frac{\nu +\gamma }{\beta }$.

$E^{0}$ is called disease-free equilibrium point (free virus equilibrium point) because there are no infectious individuals in the population, which indicates that $I = 0$ and $R = 0$. $E^{3}$ is known as endemic equilibrium point (the positive virus point) of model (4).

From the expressions of $I^{1}$ and $I^{3}$, noting that if

$$ \frac{\Lambda }{\nu }>\frac{\nu +\gamma }{\beta }, $$

the deterministic system (4) has unique positive equilibria $E^{1}$ and $E^{3}$. From this, the reproductive number of system (4) is given by

$$\begin{aligned} \xi _{0}= \frac{\beta \Lambda }{(\nu +\gamma ) \nu } . \end{aligned}$$

Similarly, at equilibrium point $E^{3}$, all the eigenvalues are nonpositive if $\xi _{0} > 1$. Hence the proposed model is globally stable if $\xi _{0} > 1$.

### Theorem 1

*The deterministic system* (4) *has*

(i) *a unique stable ‘disease*-*extinction’* (*disease*-*free equilibrium*) *equilibrium point* $E^{j}$ *for* $j=0,1,2,3$ *if* $\xi _{0} <1$. *This indicates the extinction of the disease from the population*.
(ii) *a stable positive equilibrium* $E^{j}$ *for* $j=0,1,2,3$ *exists if* $\xi _{0} >1$ *that shows the permanence of the disease*.

### Proof

The Jacobian matrix of system (4) is

$$ J= \begin{pmatrix} -\beta I-\nu & -\beta S & \sigma \\ \beta I & \beta S-(\nu +\gamma ) & 0 \\ 0 & \gamma & -(\nu +\sigma ) \end{pmatrix}. $$

Now let us show for $j=0$ ($E^{0}$), then similarly we can show for $j=1,2,3$.

The Jacobian of system (4) at $E^{0}$ gives

$$ J^{0}= \begin{pmatrix} -\nu & -\beta \frac{\Lambda }{\nu } & \sigma \\ 0 & \beta \frac{\Lambda }{\nu }-(\nu +\gamma ) & 0 \\ 0 & \gamma & -(\nu +\sigma ) \end{pmatrix}. $$

The eigenvalues are calculated as follows:

7 $$\begin{aligned} J^{E^{0}}= \begin{vmatrix} -\nu -\bar{ \lambda } & -\beta \frac{\Lambda }{\nu } & \sigma \\ 0 & \beta \frac{\Lambda }{\nu }-(\nu +\gamma )-\bar{\lambda } & 0 \\ 0 & \gamma & -(\nu +\sigma ) -\bar{\lambda } \end{vmatrix}. \end{aligned}$$

The characteristic polynomial of equation (7) is

$$ ( -\nu -\bar{\lambda }) \biggl(\beta \frac{\Lambda }{\nu }-(\nu +\gamma )- \bar{ \lambda }\biggr) \bigl(-(\nu +\sigma ) -\bar{\lambda }\bigr)=0, $$

so the eigenvalue is

$$ \bar{\lambda }=\beta \frac{\Lambda }{\nu }-(\nu +\gamma ) . $$

From the stability theory, $E^{0}$ is stable if and only if

$$\bar{\lambda }< 0, $$

or equivalently

$$ \beta \frac{\Lambda }{\nu }-(\nu +\gamma )< 0 $$

implies

$$ \xi _{0}=\beta \frac{\Lambda }{\nu (\nu +\gamma )}< 1. $$

 □

## Dynamics of the stochastic COVID-19 system

### Existence and uniqueness of the solution

To study the dynamical behavior of a dynamic biological system, the main concern is to check whether the solution of the system is uniquely global and positive. A dynamical system has a uniquely global solution if it exhibits no explosion in a given finite time. To have a uniquely global solution, the coefficients of the system must satisfy the following two conditions: (i) local Lipschitz condition, (ii) linear growth condition; see [30, 35]. However, the coefficients of the stochastic COVID-19 model (2) do not satisfy the second condition (linear growth condition), so the solution $(S_{t},I_{t},R_{t})$ of system (2) can explode in a finite time *t*. The following theorem helps us to show that there exists a unique positive solution $(S_{t},I_{t},R_{t})\in \mathbb{R}^{3}_{+}$ to COVID-19 system (2).

#### Theorem 2

*For any given initial condition* $(S_{0},I_{0},R_{0})\in \mathbb{R}^{3}_{+}$, *there is a unique nonnegative solution* $(S_{t},I_{t},R_{t})\in \mathbb{R}_{+}^{3}$ *of model* (2) *for time* $t\geq 0$.

#### Proof

The differential equation (2) has a locally Lipschitz continuous coefficient, so the model has a unique local solution $(S_{t},I_{t},R_{t})$ on $t\in [0,t_{e})$, where $t_{e}$ is the time for noise for the explosion. In order to have a global solution, we need to show that $t_{e}=\infty $ almost surely. To do this, assume that $k_{0}$ is a very large positive number $(k_{0}>0)$ so that the initial condition $(S_{0},I_{0},R_{0})\in [\frac{1}{k_{0}},k_{0} ]$. For every integer $k\geq k_{0}$, the stopping time is defined as follows:

$$\begin{aligned} \tau _{e}=\inf \biggl\{ t\in [0,t_{e}):\min (S_{t},I_{t},R_{t})\leq \frac{1}{k_{0}},\text{ or }\max (S_{t},I_{t},R_{t})\geq k\biggr\} . \end{aligned}$$

As *k* goes to ∞, $\tau _{k}$ increases. Define $\lim_{k\rightarrow \infty } \tau _{k}=\tau _{\infty }$ with $\tau _{\infty } \leq \tau _{e}$. If we can prove that $\tau _{\infty }=\infty $ almost surely, then $\tau _{e}=\infty $. If this is false, then there are two positive constants $T>0$ and $\delta \in (0,1)$ such that

$$\begin{aligned} \mathbb{P}\{\tau _{\infty }\leq T\} > \delta. \end{aligned}$$

Thus there is $k_{1}\geq k_{0}$ that satisfies

$$\begin{aligned} \mathbb{P}\{\tau _{k}\leq T\}\geq \delta,\quad k\geq k_{1}. \end{aligned}$$

Now, let us define a $C^{2}$-function *W*: $\mathbb{R}_{+}^{3} \rightarrow \mathbb{R}_{+}$ by

8 $$\begin{aligned} W(S,I,R)=\biggl(S-\alpha -\alpha \frac{\ln S}{\alpha }\biggr)+(I-1- \ln I)+ (R-1- \ln R). \end{aligned}$$

Applying Itô’s formula in Lemma 1 to Eq. (8) yields

9 $$\begin{aligned} dW(S,I,R)&=(1-\alpha /S)\,dS+\frac{(dS)^{2}}{2 S^{2}}+(1-1/I)\,dI+ \frac{(dI)^{2}}{2 I^{2}}+(1-1/S)\,dS+\frac{(dR)^{2}}{2 R^{2}} \\ &:=LW \,dt+ \bar{W}, \end{aligned}$$

where *L* is a differential operator [30].

$$\begin{aligned} \bar{W}={}&\lambda _{1} S\,dB^{1}_{t}+ \int _{\mathbb{Y}}\epsilon _{1}(y)S \bar{N}(dt,dy)-\alpha \lambda _{1} \,dB^{1}_{t}-\alpha \int _{\mathbb{Y}}\epsilon _{1}(y)\bar{N}(dt,dy) \\ &{}+\lambda _{2} I\,dB^{2}_{t}+ \int _{\mathbb{Y}}\epsilon _{2}(y)I \bar{N}(dt,dy)-\lambda _{2} \,dB^{2}_{t}- \int _{\mathbb{Y}}\epsilon _{2}(y) \bar{N}(dt,dy) \\ &{}+\lambda _{3} R\,dB^{3}_{t}+ \int _{\mathbb{Y}}\epsilon _{3}(y)R \bar{N}(dt,dy)-\lambda _{3} \,dB^{3}_{t}- \int _{\mathbb{Y}}\epsilon _{3}(y) \bar{N}(dt,dy), \end{aligned}$$

and $LW:\mathbb{R}_{+}^{3} \rightarrow \mathbb{R}_{+}$ is defined as

$$\begin{aligned} LW={}&\Lambda -\nu S+\sigma R-\alpha \frac{\Lambda }{S}+\alpha \beta I+\alpha \nu -\alpha \frac{\sigma R}{S}+ \frac{\lambda _{1}^{2}}{2}\\ &{}+ \int _{\mathbb{Y}}\epsilon _{1}^{2}(y)\pi (dy)-( \nu +\gamma ) I-\beta S+(\nu +\gamma )+ \frac{\lambda _{2}^{2}}{2} \\ &{}+ \int _{\mathbb{Y}}\epsilon _{2}^{2}(y)\pi (dy) + \gamma I-(\nu + \sigma ) R-\gamma +(\nu +\sigma ) +\frac{\lambda _{3}^{2}}{2}+ \int _{ \mathbb{Y}}\epsilon _{3}^{2}(y)\pi (dy) \\ \leq{}& \Lambda +\alpha \nu +\bigl(\alpha \beta -(\nu +\gamma )\bigr) I+( \nu + \gamma ) -\gamma +(\nu +\sigma )+\frac{\lambda _{1}^{2}}{2}+ \frac{\lambda _{2}^{2}}{2}+ \frac{\lambda _{3}^{2}}{2} \\ &{}+ \int _{\mathbb{Y}}\epsilon _{1}^{2}(y)\pi (dy)+ \int _{\mathbb{Y}} \epsilon _{2}^{2}(y)\pi (dy) + \int _{\mathbb{Y}}\epsilon _{3}^{2}(y) \pi (dy). \end{aligned}$$

By plugging in $\alpha =\frac{\nu +\gamma }{\beta }$, we get

$$\begin{aligned} LW\leq {}&\Lambda +\alpha \nu +(\nu +\gamma ) -\gamma +(\nu +\sigma )+ \frac{\lambda _{1}^{2}}{2}+ \frac{\lambda _{2}^{2}}{2}+ \frac{\lambda _{3}^{2}}{2}+ \int _{\mathbb{Y}}\epsilon _{1}^{2}(y)\pi (dy)\\ &{}+ \int _{\mathbb{Y}}\epsilon _{2}^{2}(y)\pi (dy) + \int _{\mathbb{Y}} \epsilon _{3}^{2}(y)\pi (dy) \\ :={}& C, \end{aligned}$$

where the parameter C is a positive constant. The rest of the proof follows Cai et al. [40, Lemma 2.2] and Zhu et al. [41, Theorem 1]. □

### Extinction and persistence of the disease

Since this paper considers the epidemic dynamic systems, we are focused on prevalence and persistence of COVID-19 in a population.

#### Extinction of the disease

In this subsection, we give some conditions for the extinction of COVID-19 in the stochastic COVID-19 system (2). Since the extinction of disease (epidemics) in small populations has the major challenges in population dynamics [42], it is important to study the extinction of COVID-19.

Define a parameter *ξ* as

$$\begin{aligned} \xi =\frac{\beta \Lambda }{\nu }\frac{1}{\gamma +\nu +\varphi _{2}}, \end{aligned}$$

where $\varphi _{2}=\frac{1}{2} \lambda _{2}+\int _{\mathbb{Y}}[\epsilon _{2}(y)- \ln (1+\epsilon _{2}(y))]\pi (dy)$. Here, *ξ* is the basic reproduction number for the stochastic COVID-19 model (2).

##### Remark 2

From $(e)$ and Remark 1, we have

$$\begin{aligned} \varphi _{2}&= \frac{\lambda ^{2}_{2}}{2}+ \int _{\mathbb{Y}} \bigl[ \epsilon _{2}(y)-\ln \bigl(1+ \epsilon _{2}(y)\bigr)\bigr] \pi (dy) \\ &=\frac{\lambda ^{2}_{2}}{2}+ \int _{\mathbb{Y}}\bigl[\bigl(1+\epsilon _{2}(y)\bigr)-1- \ln \bigl(1+\epsilon _{2}(y)\bigr)\bigr] \pi (dy) \\ &\geq \frac{\lambda ^{2}_{2}}{2}. \end{aligned}$$

##### Definition 1

For the stochastic model (2), if $\lim_{t\rightarrow \infty }I_{t}=0$, then the disease $I_{t}$ is said to be extinct, a.s.

##### Theorem 3

*Assume that* $(g)$ *holds*. *Then*, *for any initial condition* $(S_{0},I_{0},R_{0})\in \mathbb{R}^{3}_{+}$, *the solution* $(S_{t},I_{t},R_{t}) \in \mathbb{R}^{3}_{+}$ *of the stochastic COVID*-19 *model* (2) *has the following properties*:

$$\begin{aligned} \lim_{t\rightarrow \infty } \sup \frac{\ln I_{t}}{t}\leq \beta \frac{\Lambda }{\nu } \biggl(1-\frac{1}{\xi } \biggr),\quad \textit{a.s.} \end{aligned}$$

*If* $\xi < 1$ *holds*, *then* $I_{t}$ *can go to zero with probability one*.

*Moreover*,

$$\lim_{t\rightarrow \infty }\langle S \rangle_{t}=\frac{\Lambda }{\nu }=S_{0},\qquad \lim_{t\rightarrow \infty }\langle R \rangle_{t}=0,\quad \textit{a.s.} $$

##### Proof

Integrating both sides of model (2) and dividing by *t* gives

10 $$\begin{aligned} & \frac{S_{t}-S_{0}}{t}=\Lambda -\beta \langle S \rangle_{t} \langle I \rangle_{t}-\nu \langle S \rangle_{t}+ \sigma \langle R \rangle_{t}+\frac{\lambda _{1}}{t} \int _{0}^{t}S_{r}\,dB^{1}_{r} \\ &\phantom{\frac{S_{t}-S_{0}}{t}=}{}+ \frac{1}{t} \int _{0}^{t} \int _{\mathbb{Y}}\epsilon _{1}(y) S_{r} \bar{N}(dr,dt), \end{aligned}$$

11 $$\begin{aligned} &\frac{I_{t}-I_{0}}{t}=\beta \langle S \rangle_{t} \langle I \rangle_{t}-(\gamma +\nu )\langle I \rangle_{t}+ \frac{\lambda _{2}}{t} \int _{0}^{t}I_{r}\,dB^{2}_{r}+ \frac{1}{t} \int _{0}^{t} \int _{\mathbb{Y}}\epsilon _{2}(y) I_{r} \bar{N}(dr,dt), \end{aligned}$$

12 $$\begin{aligned} & \frac{R_{t}-R_{0}}{t}=\gamma \langle I \rangle_{t}-(\nu + \sigma )\langle R \rangle_{t}+ \frac{\lambda _{3}}{t} \int _{0}^{t}R_{r}\,dB^{3}_{r}+ \frac{1}{t} \int _{0}^{t} \int _{\mathbb{Y}}\epsilon _{3}(y) R_{r} \bar{N}(dr,dt). \end{aligned}$$

Multiplying both sides of Eq. (12) by $\frac{\sigma }{\nu +\sigma }$, we have

13 $$\begin{aligned} \frac{\sigma }{\nu +\sigma }\frac{R_{t}-R_{0}}{t}={}& \frac{\sigma }{\nu +\sigma } \gamma \langle I \rangle_{t}-\sigma \langle R \rangle_{t}+ \frac{\sigma }{\nu +\sigma }\frac{\lambda _{3}}{t} \int _{0}^{t}R_{r}\,dB^{3}_{r} \\ &{}+ \frac{\sigma }{\nu +\sigma }\frac{1}{t} \int _{0}^{t} \int _{\mathbb{Y}} \epsilon _{3}(y) R_{r} \bar{N}(dr,dt). \end{aligned}$$

Adding Eqs. (10), (11), and (13), we obtain

14 $$\begin{aligned} &\frac{S_{t}-S_{0}}{t}+ \frac{I_{t}-I_{0}}{t}+ \frac{\sigma }{\nu +\sigma } \frac{R_{t}-R_{0}}{t} \\ &\quad=\Lambda -\nu \langle S \rangle_{t}+ \frac{\lambda _{1}}{t} \int _{0}^{t}S_{r}\,dB^{1}_{r}+ \frac{1}{t} \int _{0}^{t} \int _{\mathbb{Y}}\epsilon _{1}(y) S_{r} \bar{N}(dr,dt) \\ &\qquad{}-(\gamma +\nu )\langle I \rangle_{t}+\frac{\lambda _{2}}{t} \int _{0}^{t}I_{r}\,dB^{2}_{r}+ \frac{1}{t} \int _{0}^{t} \int _{\mathbb{Y}}\epsilon _{2}(y) I_{r} \bar{N}(dr,dt) \\ &\qquad{}\times\frac{\sigma }{\nu +\sigma } \gamma \langle I \rangle_{t}+ \frac{\sigma }{\nu +\sigma } \frac{\lambda _{3}}{t} \int _{0}^{t}R_{r}\,dB^{3}_{r}+ \frac{\sigma }{\nu +\sigma }\frac{1}{t} \int _{0}^{t} \int _{\mathbb{Y}} \epsilon _{3}(y) R_{r} \bar{N}(dr,dt) \\ &\quad=\Lambda -\nu \langle S \rangle_{t}- \biggl((\gamma +\nu )- \frac{\sigma }{\nu +\sigma } \gamma \biggr)\langle I \rangle \\ &\qquad{}+\frac{\lambda _{1}}{t} \int _{0}^{t}S_{r}\,dB^{1}_{r}+ \frac{1}{t} \int _{0}^{t} \int _{\mathbb{Y}}\epsilon _{1}(y) S_{r} \bar{N}(dr,dt) \\ &\qquad{}+\frac{\lambda _{2}}{t} \int _{0}^{t}I_{r}\,dB^{2}_{r}+ \frac{1}{t} \int _{0}^{t} \int _{\mathbb{Y}}\epsilon _{2}(y) I_{r} \bar{N}(dr,dt) \\ &\qquad{}+\frac{\sigma }{\nu +\sigma }\frac{\lambda _{3}}{t} \int _{0}^{t}R_{r}\,dB^{3}_{r}+ \frac{\sigma }{\nu +\sigma }\frac{1}{t} \int _{0}^{t} \int _{\mathbb{Y}} \epsilon _{3}(y) R_{r} \bar{N}(dr,dt). \end{aligned}$$

Rewrite Eq. (14) as

15 $$\begin{aligned} \langle S \rangle_{t}=\frac{\Lambda }{\nu }- \biggl( \frac{\gamma +\nu +\sigma }{\nu +\sigma } \biggr)\langle I \rangle_{t}+\bar{\Phi }_{t}, \end{aligned}$$

where

$$\begin{aligned} \bar{\Phi }_{t}={}&{-}\frac{1}{\nu } \biggl( \frac{S_{t}-S_{0}}{t}+ \frac{I_{t}-I_{0}}{t}+ \frac{\sigma }{\nu +\sigma } \frac{R_{t}-R_{0}}{t} \biggr) \\ &{}+ \frac{1}{\nu } \biggl( \frac{\lambda _{1}}{t} \int _{0}^{t}S_{r}\,dB^{1}_{r} +\frac{1}{t} \int _{0}^{t} \int _{\mathbb{Y}}\epsilon _{1}(y) S_{r} \bar{N}(dr,dt) \biggr) \\ &{}+\frac{1}{\nu } \biggl(\frac{\lambda _{2}}{t} \int _{0}^{t}I_{r}\,dB^{2}_{r}+ \frac{1}{t} \int _{0}^{t} \int _{\mathbb{Y}}\epsilon _{2}(y) I_{r} \bar{N}(dr,dt) \biggr) \\ &{}+\frac{1}{\nu } \biggl(\frac{\sigma }{\nu +\sigma } \frac{\lambda _{3}}{t} \int _{0}^{t}R_{r}\,dB^{3}_{r}+ \frac{\sigma }{\nu +\sigma }\frac{1}{t} \int _{0}^{t} \int _{\mathbb{Y}} \epsilon _{3}(y) R_{r} \bar{N}(dr,dt) \biggr). \end{aligned}$$

From Lemma 2(i)–(ii),

16 $$\begin{aligned} \lim_{t\rightarrow \infty } \bar{\Phi }_{t}=0,\quad \text{a.s.} \end{aligned}$$

Therefore, Eq. (15) becomes

17 $$\begin{aligned} \langle S \rangle_{t}=\frac{\Lambda }{\nu }- \biggl( \frac{\gamma +\nu +\sigma }{\nu +\sigma } \biggr)\langle I \rangle_{t}. \end{aligned}$$

Setting $Z=\ln I_{t}$ and applying Itô’s formula to *Z* yields

18 $$\begin{aligned} dZ={}& d \ln I_{t}=\frac{1}{I_{t}}\,dI_{t}- \frac{1}{2I^{2}_{t}}[dI_{t}]^{2} \\ ={}&\bigl(\beta S_{t}-(\nu +\gamma )-\varphi _{2}\bigr)\,dt+ \lambda _{2} I_{t}\,dB^{2}_{t}+ \int _{\mathbb{Y}}\ln \bigl(1+\epsilon _{2}(y)\bigr) \bar{N}(dt,dy) . \end{aligned}$$

Integrating both sides of Eq. (18) and dividing by *t* gives

19 $$\begin{aligned} \frac{\ln I_{t}}{t}= \beta \langle S \rangle_{t}-(\nu + \gamma )-\varphi _{2}+ \frac{\lambda _{2} I_{t}\,dB^{2}_{t}}{t}+\frac{1}{t} \int _{\mathbb{Y}} \ln \bigl(1+\epsilon _{2}(y)\bigr) \bar{N}(dt,dy)+\frac{\ln I_{0}}{t}. \end{aligned}$$

Upon plugging in $\langle S \rangle_{t}$ of Eq. (17) into Eq. (19), we get

20 $$\begin{aligned} \frac{\ln I_{t}}{t}={}& \beta \biggl(\frac{\Lambda }{\nu }- \biggl( \frac{\gamma +\nu +\sigma }{\nu +\sigma } \biggr)\langle I \rangle_{t} \biggr)-(\nu + \gamma )- \varphi _{2}+\frac{\lambda _{2} I_{t}\,dB^{2}_{t}}{t} \\ &{}+ \frac{1}{t} \int _{\mathbb{Y}}\ln \bigl(1+\epsilon _{2}(y)\bigr) \bar{N}(dt,dy)+ \frac{\ln I_{0}}{t} \\ ={}& \beta \frac{\Lambda }{\nu }-(\nu +\gamma )-\varphi _{2}-\beta \biggl( \frac{\gamma +\nu +\sigma }{\nu +\sigma } \biggr))\langle I \rangle_{t}+ \frac{\lambda _{2} I_{t}\,dB^{2}_{t}}{t} \\ &{}+ \frac{1}{t} \int _{\mathbb{Y}} \ln \bigl(1+\epsilon _{2}(y)\bigr) \bar{N}(dt,dy)+\frac{\ln I_{0}}{t} \\ ={}& \beta \frac{\Lambda }{\nu }-(\nu +\gamma +\varphi _{2})-\beta \biggl( \frac{\gamma +\nu +\sigma }{\nu +\sigma } \biggr)\langle I \rangle_{t}+ \frac{\lambda _{2} I_{t}\,dB^{2}_{t}}{t}+ \frac{\psi _{2}(t)}{t}+ \frac{\ln I_{0}}{t} \\ \leq{}& \beta \frac{\Lambda }{\nu } \biggl(1-\frac{\nu }{\beta \Lambda }( \nu +\gamma +\varphi _{2}) \biggr)-\beta \biggl( \frac{\gamma +\nu +\sigma }{\nu +\sigma } \biggr)\langle I \rangle_{t}+ \frac{\lambda _{2} I_{t}\,dB^{2}_{t}}{t}+\frac{\psi _{2}(t)}{t}+ \frac{\ln I_{0}}{t} \\ \leq{}& \beta \frac{\Lambda }{\nu } \biggl(1-\frac{1}{\xi } \biggr)- \beta \biggl(\frac{\gamma +\nu +\sigma }{\nu +\sigma } \biggr)\langle I \rangle_{t}+ \frac{\lambda _{2} I_{t}\,dB^{2}_{t}}{t}+\frac{\psi _{2}(t)}{t}+ \frac{\ln I_{0}}{t} \\ \leq{}& \beta \frac{\Lambda }{\nu } \biggl(1-\frac{1}{\xi } \biggr)- \beta \biggl(\frac{\gamma +\nu }{\nu +\sigma } \biggr)\langle I \rangle_{t}+ \frac{\lambda _{2} I_{t}\,dB^{2}_{t}}{t}+\frac{\psi _{2}(t)}{t}+ \frac{\ln I_{0}}{t}, \\ & \text{since }- \frac{\gamma +\nu +\sigma }{\nu +\sigma }< - \frac{\gamma +\nu }{\nu +\sigma }. \end{aligned}$$

From $(f)$ and the theorem of large numbers [43] we have

21 $$\begin{aligned} \lim_{t\rightarrow \infty }\frac{\psi _{2}(t)}{t}=0,\quad \text{a.s.} \end{aligned}$$

and

22 $$\begin{aligned} \lim_{t\rightarrow \infty }\frac{B_{t}}{t}=0,\quad \text{a.s.} \end{aligned}$$

By applying the superior limit ($\lim_{t\rightarrow \infty } \sup $) on both sides of Eq. (20) gives

23 $$\begin{aligned} \lim_{t\rightarrow \infty } \sup \frac{\ln I_{t}}{t} &\leq \beta \frac{\Lambda }{\nu } \biggl(1-\frac{1}{\xi } \biggr),\quad \text{a.s.} \end{aligned}$$

If $\xi <1$ holds, then $\beta \frac{\Lambda }{\nu } (1-\frac{1}{\xi } )<0$.

Therefore,

24 $$\begin{aligned} \lim_{t\rightarrow \infty }I_{t}=0. \end{aligned}$$

From Definition 1, this implies that $I_{t}$ can tend to zero with probability one. Similarly, we can show that

25 $$\begin{aligned} \lim_{t\rightarrow \infty }\langle R \rangle_{t}=0. \end{aligned}$$

Recall Eq. (6),

$$\begin{aligned} X=\frac{\Lambda }{\nu }+X_{0} e^{-\nu t}. \end{aligned}$$

Using Eqs. (24) and (25), and

$$\begin{aligned} \lim_{t\rightarrow \infty }X=\lim_{t\rightarrow \infty }(S_{t}+I_{t}+R_{t})= \frac{\Lambda }{\nu }, \end{aligned}$$

we obtain

$$\begin{aligned} \lim_{t\rightarrow \infty }\langle S \rangle_{t}= \frac{\Lambda }{\nu }=S_{0} . \end{aligned}$$

 □

#### Persistence of the disease

This section deals with the persistence in mean of the disease in model (2). Before we state the theorem, we define persistence in mean.

##### Definition 2

If $\lim_{t\rightarrow \infty }\langle S \rangle_{t} >0$, $\lim_{t\rightarrow \infty }\langle I \rangle_{t} >0$, $\lim_{t\rightarrow \infty }\langle R \rangle_{t} >0$, almost surely, then we can say that system (2) is persistent in mean.

##### Theorem 4

*For given initial values* $(S_{0},I_{0},R_{0})\in \mathbb{R}_{+}^{3}$, *the solution* $(S_{t},I_{t},R_{t})\in \mathbb{R}_{+}^{3}$ *of model* (2) *exists when* $\xi >1$,

$$\lim_{t\rightarrow \infty }\langle S \rangle_{t}=\tilde{S}, \qquad \lim _{t\rightarrow \infty }\langle I \rangle_{t}=\tilde{I},\qquad \lim _{t\rightarrow \infty }\langle R \rangle_{t}=\tilde{R}, \quad\textit{a.s.},$$

*where*

$$\begin{aligned} &\tilde{S}=\frac{\Lambda }{\nu }- \frac{\gamma +\nu +\sigma }{\gamma +\nu } \frac{\Lambda }{\nu } \biggl(1- \frac{1}{\xi } \biggr), \qquad\tilde{I}=\frac{\nu +\sigma }{\gamma +\nu } \frac{\Lambda }{\nu } \biggl(1- \frac{1}{\xi } \biggr), \\ &\tilde{R}=\frac{1}{\gamma +\nu } \frac{\Lambda }{\nu } \biggl(1- \frac{1}{\xi } \biggr). \end{aligned}$$

##### Proof

Recall Eq. (20)

26 $$\begin{aligned} \frac{\ln I_{t}}{t}= \beta \frac{\Lambda }{\nu } \biggl(1- \frac{1}{\xi } \biggr)-\beta \biggl(\frac{\gamma +\nu }{\nu +\sigma } \biggr)\langle I \rangle_{t}+\frac{\lambda _{2} I_{t}\,dB^{2}_{t}}{t}+ \frac{\psi _{2}(t)}{t}+\frac{\ln I_{0}}{t}, \end{aligned}$$

or equivalently,

27 $$\begin{aligned} \beta \biggl(\frac{\gamma +\nu }{\nu +\sigma } \biggr)\langle I \rangle_{t}= - \frac{\ln I_{t}}{t}+\beta \frac{\Lambda }{\nu } \biggl(1- \frac{1}{\xi } \biggr)+\frac{\lambda _{2}}{t} I_{t}\,dB^{2}_{t}+ \psi _{2}(t)+ \frac{\ln I_{0}}{t}. \end{aligned}$$

From Lemma 2 and Eqs. (16), (21), and (22), we get

28 $$\begin{aligned} \lim_{t\rightarrow \infty } \langle I \rangle_{t}= \frac{\nu +\sigma }{\gamma +\nu } \frac{\Lambda }{\nu } \biggl(1-\frac{1}{\xi } \biggr)= \tilde{I}, \quad\text{a.s.} \end{aligned}$$

Substituting Eq. (28) into Eq. (17), and taking limit on both sides, yields

29 $$ \lim_{t\rightarrow \infty } \langle S \rangle_{t}= \frac{\Lambda }{\nu }- \frac{\gamma +\nu +\sigma }{\gamma +\nu } \frac{\Lambda }{\nu } \biggl(1- \frac{1}{\xi } \biggr)=\tilde{S}. $$

Furthermore, applying $\lim_{t\rightarrow \infty }$ to Eq. (12) and replacing $\langle I \rangle_{t}$ by Eq. (28) yields

30 $$\begin{aligned} \lim_{t\rightarrow \infty }\langle R \rangle_{t}= \frac{1}{\gamma +\nu } \frac{\Lambda }{\nu } \biggl(1-\frac{1}{\xi } \biggr)= \tilde{R}. \end{aligned}$$

The proof is complete. □

##### Remark 3

From Theorems 3 and 4, we can take the value of *ξ* as the threshold of system (2). The value of *ξ* indicates the prevalence and extinction of COVID-19. Here, we can observe that the Gaussian and jump noises have a significant effect on the behavior of system (2).

## Discussion and numerical experiments

This section deals with the theoretical results of the investigated deterministic and stochastic epidemic systems by applying numerical simulations. Here, to find out the impact of Gaussian and non-Gaussian noise intensities on this epidemic dynamics, we compare the trajectories of the deterministic and stochastic systems. We choose the initial value $(S_{0},I_{0},R_{0})=(70,50,20), \Lambda =0.0072, \beta =0.002, \sigma =0.01, \gamma =0.02$. The other values of the parameters are given in the figures.

**Figure 1:**
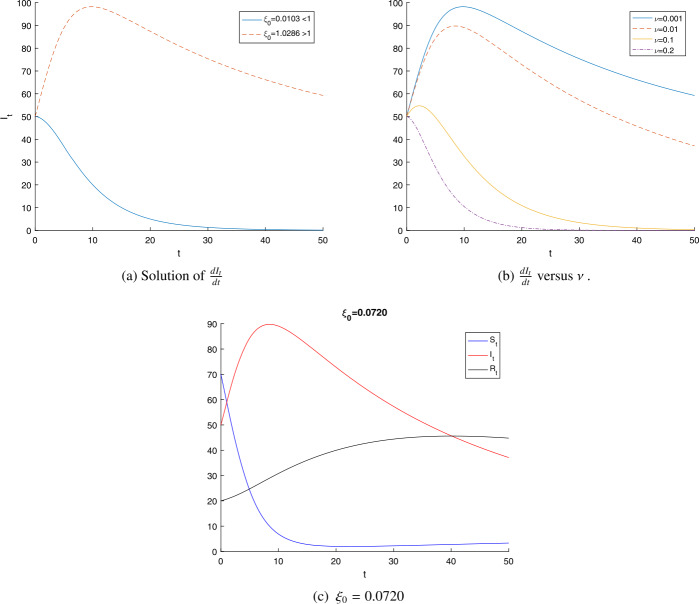
Sample path of $\frac{dI}{dt}$ (**a**) when $\xi _{0}=1.0286$ and $\xi _{0}=0.0103$. (**b**) The phaseline of $dI_{t}/dt$ at different values of *ν*. (**c**) When the reproduction number $\xi _{0} <1$

Figure 1 plots the numerical simulation of the deterministic epidemic model (4). Figure 1(a) shows the results of Theorem 1 for different values of the reproductive number $\xi _{0}$. We can easily see from the results that the infectious disease of system (4) goes to extinction for $\xi _{0} <1$, almost surely, whereas the disease persists if $\xi _{0} >1$. The parameter *ν* will lead to a decrease in $\xi _{0}$. This tells us that the extinction of the disease is very fast as *ν* increases, this phenomenon is plotted in Fig. 1(b). As *ν* increases, the value of $\xi _{0}$ is less than one, thus, according to Theorem 1, asymptotically results into extinction of COVID-19 in the population, i.e., $I_{t}$ can go to zero with probability one. The phase line of the COVID-19 epidemic model (4) is given in Fig. 1(c) when $\xi _{0} <1$ and $\nu =0.01$.

**Figure 2:**
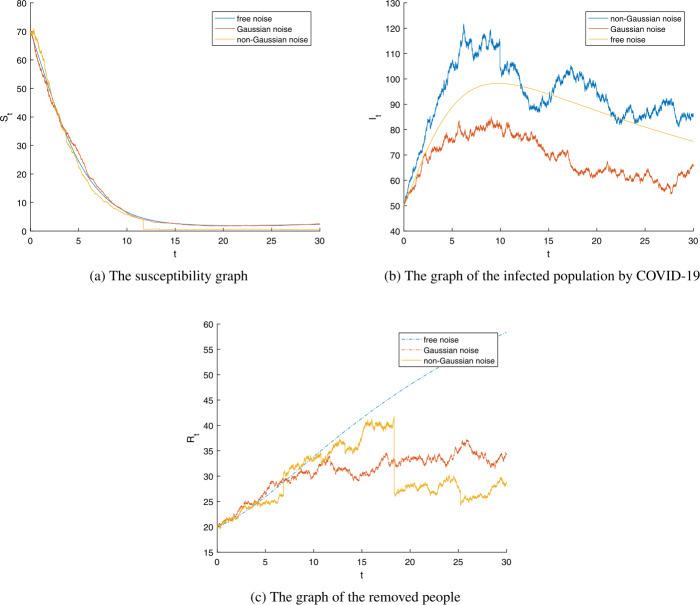
The numerical results of model (2). (**a**) The graph of the susceptible. (**b**) The graph of the infected people.(**c**) The graph of the recovered people. Parameters $S_{0}=70$, $I_{0}=50$, $R_{0}=20$, $\Lambda =0.0072$, $\beta =0.002$, $\nu =0.001$, $\sigma =0.01$, $\gamma =0.02$, $\lambda _{j}=0.047$, $\epsilon _{j}(y)=0.004, j=1,2,3, \xi =0.9760 <1$.

**Figure 3:**
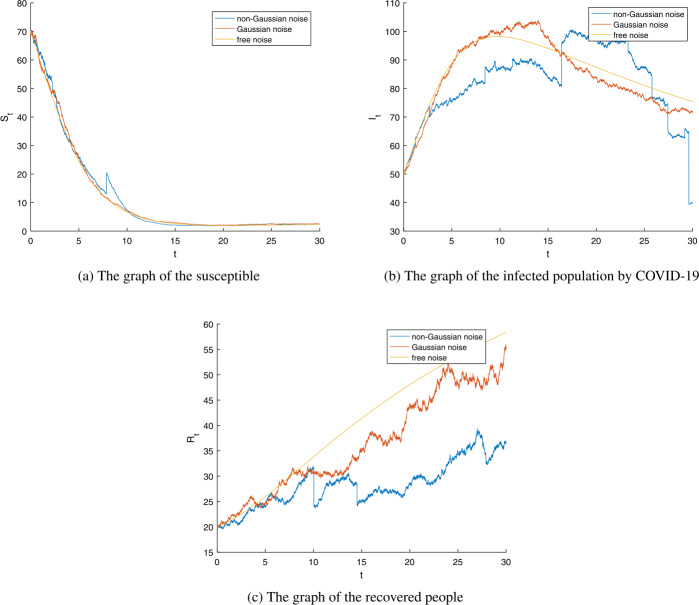
The numerical simulation of model (2). (**a**) The graph of the susceptible. (**b**) The graph of the infected people. (**c**) The graph of the recovered people from COVID-19. Parameters $S_{0}=70$, $I_{0}=50$, $R_{0}=20$, $\Lambda =0.0072$, $\beta =0.002$, $\nu =0.001$, $\sigma =0.01$, $\gamma =0.02$, $\lambda _{1}=0.047$, $\lambda _{2}=0.019$, $\lambda _{3}=0.047$, $\epsilon _{j}(y)=0.004$, $j=1,2,3$, $\xi =1.02 > 1$

In Figs. 2 and 3, we fixed the parameters $\nu =0.001$, $\epsilon _{j}(y)=0.004$ for $j=1,2,3$, and $\mathbb{Y}=(0,\infty ), \pi (\mathbb{Y})=1$. Here, the value of the basic reproductive number $\xi _{0}$ is 1.0286, and $\xi =0.9349$. Having these values, the solution $(S_{t},I_{t},R_{t})$ of system (2) satisfies the property in Theorem 3, i.e.,

$$\begin{aligned} \lim_{t\rightarrow \infty }\frac{\ln I_{t}}{t}\leq \beta \frac{\Lambda }{\nu } \biggl(1-\frac{1}{\xi } \biggr) =-0.0015 < 0 \quad\text{a.s.} \end{aligned}$$

This shows that $I_{t}$ can vanish as *t* goes to infinity. This happens because of the Lévy noise effect. When $\lambda _{2}=0.019$ and $\xi =1.0093$, the solution $(S_{t},I_{t},R_{t})$ of model (2) satisfies the condition in Theorem 4. This scenario means that

$$\begin{aligned} &\lim_{t\rightarrow \infty } \langle S \rangle_{t}=7.1025, \\ &\lim_{t\rightarrow \infty } \langle I \rangle_{t}=0.0346, \end{aligned}$$

and

$$\begin{aligned} \lim_{t\rightarrow \infty }\langle R \rangle_{t}=3.1460,\quad \text{a.s.} \end{aligned}$$

**Figure 4:**
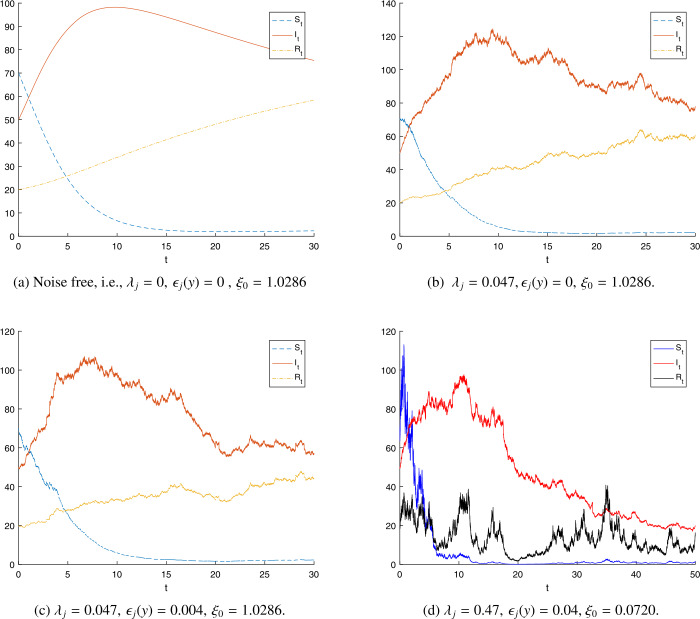
This figure shows the numerical simulation of the stochastic COVID-19 model (2) with $S_{0}=70$, $I_{0}=50$, $R_{0}=20$, $\Lambda =0.0072$, $\beta =0.002$, $\nu =0.001$, $\sigma =0.01$, $\gamma =0.02$, $\xi =0.9284 <1$, $j=1,2,3$

This numerical experiment shows that COVID-19 will prevail. Note that Fig. 2 and Fig. 3 only differ by the value of $\lambda _{2}$. The relationship of the variables $S_{t}, I_{t}$, and $R_{t}$ is plotted in Fig. 4. When the reproductive number $\xi _{0}$ is less than 1, the stochastic reproductive number *ξ* is also less than 1. For this case, the sample paths of the stochastic COVID-19 model are plotted in Figs. 4(b), 4(c), and 4(d).

The numerical solutions imply that reducing contact rate, washing hands, improving treatment rate, and environmental sanitation are the most crucial activities to eradicate the COVID-19 disease from the community.

## Conclusion

The non-Gaussian noise plays a significant role in evolution of epidemic dynamical processes like HIV, SARS, avian influenza, and so on. In this work, we have studied the stochastic COVID-19 epidemic model driven by both Gaussian and non-Gaussian noises. In Theorem 2, we proved that model (2) has a unique nonnegative solution. We also investigated some conditions for the extinction and persistence during the COVID-19 epidemic. We have applied a matlab program to study the behavior of the solution of the model. We have illustrated with numerical results the changing impact of the noise intensities and the parameter *ν* on the number of infectious individuals. The results established in the present study can be used to examine dynamical behaviors for COVID-19, HIV, SARS, and so on.

By using the Euler–Maruyama (EM) method [36, 37], we gave some numerical solutions to illustrate the extinction and persistence of the disease in the deterministic system and stochastic counterparts for comparison. We also obtained and compared the basic reproduction numbers for the deterministic model as well as the stochastic one. From the comparison, we observed that the basic reproduction number of the stochastic COVID-19 model is much smaller than that of the deterministic COVID-19 model; this shows that the stochastic approach is more realistic than the deterministic one. In other words, the jump noise and white noise can change the behavior of the model. The noises can force COVID-19 (disease) to become extinct.

Furthermore, we showed that the disease can go to extinction if $\xi <1$, while COVID-19 becomes persistent for $\xi >1$; see Theorems 3 and 4.

From the findings, we concluded that if $\xi <1$, it is possible that the spread of the disease can be controlled, but for $\xi >1$, COVID-19 can be persistent. $\frac{\beta \Lambda }{\nu }\geq \varphi _{2}$ implies that the Gaussian and non-Gaussian noises are small. From this result, we conclude that efforts should be encouraged in order to achieve a disease-free population.

## Data Availability

The authors confirm that the data supporting the findings of this study are available within the articles cited therein.
